# RETRACTED ARTICLE: Expression of integrin genes and proteins in progression and dissemination of colorectal adenocarcinoma

**DOI:** 10.1186/1472-6890-13-16

**Published:** 2013-05-24

**Authors:** Marcos VA Denadai, Luciano S Viana, Renato J Affonso Jr, Sandra R Silva, Indhira D Oliveira, Silvia R Toledo, Delcio Matos

**Affiliations:** 1 Interdisciplinary Surgery, Federal University of São Paulo, São Paulo, SP Brazil; 2 Barretos Cancer Hospital, Fundação Pio XII, Barretos, SP Brazil; 3 Genetics Laboratory (GRAACC), Federal University of São Paulo, São Paulo, SP Brazil

**Keywords:** Integrin, Extracellular matrix, Colorectal carcinoma

## Abstract

**Background:**

This study aimed to evaluate the relationship between the expression levels of selected integrin genes and proteins and cell differentiation, TNM stage, histological type and other variables potentially associated with the progression and dissemination of colorectal carcinoma (CRC).

**Methods:**

A total of 114 patients (63 men and 51 women) were treated for CRC between 2006 and 2009, including 25 (21.9%) TNM I, 39 (34.2%) TNM II, 34 (29.8%) TNM III, and 16 (14.1%) TNM IV. Regarding grade, 91 (79.8%) were grade II, 14 (12.2%) were grade III and nine (7.8%) were grade I. Reverse-transcription polymerase chain reaction (RT-PCR) and tissue microarray (TMA) methods were used to examine the expression levels of the genes *ITGAV*, *ITGA3*, *ITGA5*, *ITGB5*, and *ITGA6*, and their proteins, respectively.

**Results:**

In relation to TNM staging, *ITGB5* and *ITGA3* were over-expressed in stages III versus I. These results were confirmed by TMA analysis. In terms of age, *ITGA5* was under-expressed according to RT-PCR, but over-expressed by TMA in patients over 60 years, while *ITGA5* gene and protein levels were increased in mucinous carcinomas. In addition *ITGAV* gene and protein levels were elevated in tumors with neural invasion, and *ITGA6* gene and protein were over-expressed in cases with venous invasion. All these results were significant at *P* < 0.05.

**Conclusion:**

The results of this study suggest that over-expression of some integrins is associated with TNM III stage, increased risk of vascular and neural invasion, and mucinous histology in patients with CRC.

**Electronic supplementary material:**

The online version of this article (doi:10.1186/1472-6890-13-16) contains supplementary material, which is available to authorized users.

## Background

The increasing availability of molecular biology tools has revealed the coexistence of numerous processes during carcinogenesis, from imbalances in the cell cycle to the development of a neoplastic tissue with invasive characteristics. Extracellular matrix (ECM) proteins interact directly with cell surface receptors/adhesion molecules to initiate signal transduction pathways and modulate different processes [1] that participate in various cellular events such as adhesion, migration, proliferation, cell differentiation, apoptosis and angiogenesis [2]. Integrins appear to act as adhesion receptors for ECM proteins such as collagen, laminin and fibronectin, and also play a role in cell–cell adhesion [3].

Integrins display a heterodimeric structure composed of an α subunit, with a large extracellular domain containing various regions with cationic links and a short intracellular domain, and a β subunit, which has a large extracellular domain with repeated sequences of amino acids, containing a large number of cysteine residues. To date, 18 α subunits and eight β subunits have been identified [4, 5]. The majority of integrin binding occurs at its extracellular domain, with the peptide sequence Arg-Gly-Asp, which is present in many ECM proteins [6, 7], acting as a transmembrane connector between the extracellular ligand and the cytoplasmic environment, thus participating in bidirectional signaling by different cell types [8].

The role of integrins is to modulate adhesion phenomena that are implicated in processes such as cell growth and development, apoptosis, adhesion, migration, invasion, phagocytosis and cell morphology [9–11]. Studies by Von Lampe et al. [12] showed that the expression levels of α3 and α5 integrins were very low in adenomas, and absent in the majority of colorectal carcinomas (CRCs). In contrast, the α6 integrin maintains its expression in adenomas, but its expression levels are very low in malignancies with infiltrative growth characteristics, suggesting an association with CRC progression [12].

Some studies [13] have reported a crucial role for the αV integrin in the migration of cells in the colon, but the dynamics of this integrin and its effects are still poorly understood. Some integrins show different expression profiles during tumor growth and progression, suggesting their potential as targets for the diagnosis and therapy of cancer [14–16].

## Methods

### Clinical characteristics

This study included patients of either gender aged ≥18 years who underwent surgery at the Colorectal Surgery Department, Barretos Cancer Hospital, Brazil, between 2006 and 2009, and who had cryopreserved tumor samples obtained during surgery and paraffin-embedded tissue available for further histopathological analysis. Patients who had received neoadjuvant treatment (chemotherapy or radiotherapy), patients in whom the primary CRC site had not been removed, and patients with a previous or current diagnosis of other primary malignancies in any location of the body, other than non-melanoma skin cancer or cervical carcinoma *in situ*, were excluded from the study. A total of 114 patients with colon cancer (63 men, 51 women) were therefore included. Their median age was 54.5 years (range, 24–85 years). This study was approved by the Barretos Cancer Hospital Ethics Committee, São Paulo, Brazil. Project number: 128/2008.

The histological characteristics commonly associated with tumor dissemination and progression were categorized as follows: venous invasion (presence vs. absence), lymphatic vessel invasion (presence vs. absence), perineural invasion (presence vs. absence), lymph node metastasis (presence vs. absence), distant metastases (presence vs. absence), and TNM grouping ( I vs. II, I vs. III, I vs. IV , I: control group) (AJCC 2002, 6th edition).

To test the hypothesis that integrins might be associated with CRC progression and dissemination, we examined differences in their gene and protein expression levels with respect to the histological covariates mentioned above, using both reverse-transcription polymerase chain reaction (RT-PCR) and the immunohistochemical (IHC) tests using the tissue microarray technique (TMA). The use of human tissue for research was approved by the Institutional Review Board, and the design of this study followed the principles of the Helsinki Declaration and complied with the principles of good clinical practice. The clinical characteristics of the patients are presented in Table 1.

### Tumor specimens

Cryopreserved samples were embedded in medium for frozen tissue specimens (Tissue-Tek OCT; Sakura Finetek, Torrance, Calif., USA) and fitted into a cryostat (CM1850 UV; Leica Microsystems, Nussloch, Germany) for histological analysis. Slides mounted with sections of 4 μm thickness were subjected to the hematoxylin-eosin staining technique (Merck, Darmstadt, Germany) and then analyzed by a pathologist to ensure that the selected samples represented the general histology of the tumor and were free of necrosis or calcifications. Areas of interest were identified microscopically and marked for macrodissection. These slides were used as ‘guides’ to select and cut tissues in the cryostat. For each sample, sterile individual scalpel blades were used. After discarding inappropriate areas for RNA extraction, the tissue was mechanically macerated with liquid nitrogen and transferred to 1.5-ml microtubes, which were RNase and DNase free and contained 1,000 μl TRIzol (Invitrogen, Carlsbad, Calif., USA). RNA was extracted according to the manufacturer’s instructions, and RNA quantification was performed using a spectrophotometer (Thermo Scientific NanoDrop 2000). The quality and integrity of the RNA were verified by the presence of 28S and 18S bands in agarose gel and stained with 1% ethidium bromide to assure the absence of degradation of the RNA samples.

**Table 1:** Clinical characteristics of patients

Parameter		Number (%)
Age (yr)	<60	56
	>60	58
Sex	Male	63 (55.3%)
	Female	51 (44.7%)
Tumor location	Colon	82 (71.9%)
	Rectum	32 (28.1%)
Differentiation	Well	9 (7.8%)
	Moderate	91 (79.8%)
	Poor	14 (12.2%)
Histologic type	Adenocarcinoma	97 (85.1%)
	Mucinous carcinoma	17 (14.9%)
Histological characteristics	Inflammatory infiltrate	93 (81.6%)
	Perineural invasion	8 (7.1%)
	Vascular invasion	21 (18.4%)
TNM staging	I	25 (21.9%)
	II	39 (34.2%)
	III	34 (29.8%)
	IV	16 (14.1%)

**Figure 1:**
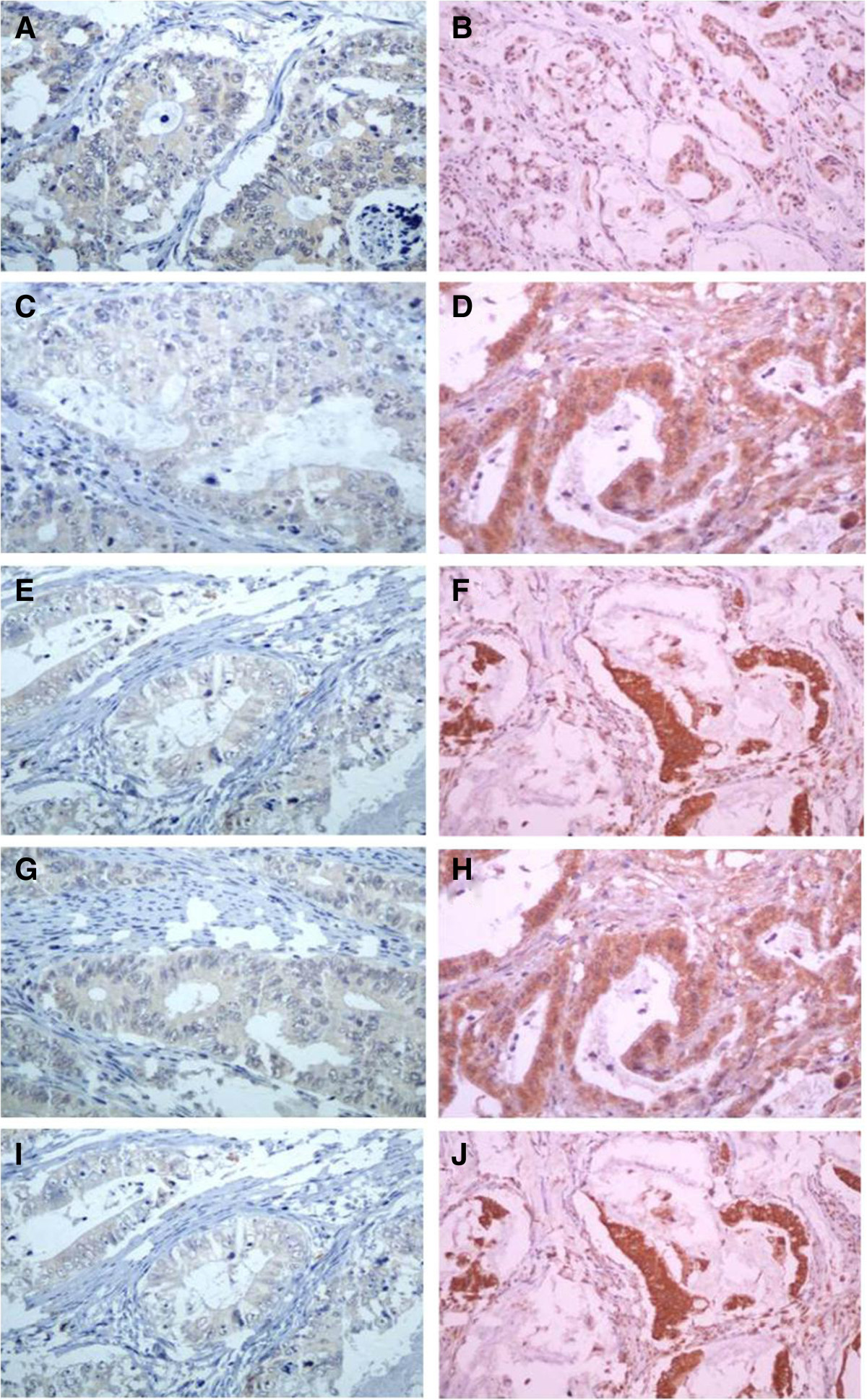
**Expression of integrins protein in CRC by IHC staining.** ( X 400). **A** , **C**, **E**, **G**, **I**, Low expression of α3, α5 , α6, αv, β5 integrins protein, respectively. **B**, **D**, **F**, **H** , **J**, Overexpression of α3, α5, α6, αv, β5 integrins protein respectively.

RNA was purified with the RNeasy mini kit (Qiagen, Valencia, Calif., USA) following the manufacturer’s recommendations, eluted with 30 ml of water free of RNase and DNase (Qiagen), quantified spectrophotometrically at a wavelength of 260 nm (NanoVue; GE Healthcare, Chicago, Ill., USA) and stored at -80°C until use. RT-PCR was performed using the Super- Script™ III first-strand synthesis SuperMix (Invitrogen), as recommended by the manufacturer. The reaction was carried out in a 20 μl final volume containing 2 μg of total RNA with oligo(dT)_20_ as a primer. The transcription phase was carried out in a thermal cycler (Mastercycler® ep Gradient S; Eppendorf, Hamburg, Germany), and the cDNA was stored at -20°C for future reactions.

**Table 2:** Analysis of the expression of genes according to the categorization of covariates with descriptions of the fold regulation and statistical differences (Mann–Whitney U test; p values are shown in parentheses) in the study cohort (n = 114)

Covariates	Comparison performed	Genes				
	Control vs. test group	*ITGA3*	*ITGA5*	*ITGA6*	*ITGB5*	*ITGAV*
		FR ( p )	FR ( p )	FR ( p )	FR ( p )	FR ( p )
Gender	Female vs. male	- 1.81 (0.377)	1.20 (0.467)	1.22 (0.599)	1.04 (0.360)	1.17 (0.068)
Age	<60 vs. ≥60 years	1.27 (0.301)	***-1.54 (0.016)***	1.15 (0.524)	1.14 (0.267)	-1.22 (0.128)
Histological classification	Adeno. vs. mucinous	4.02 (0.700)	***1.25 (0.029)***	-1.05 (0.358)	1.34 (0.815)	-1.02 (0.983)
Tumor grading	Low vs. high grade	2.94 (0.752)	1.88 (0.05)	-1.34 (0.098)	1.36 (0.889)	1.08 (0.371)
Venous invasion	Absent vs. present	1.78 (0.653)	-1.13 (0.510)	***1.42 (0.047)***	1.12 (0.718)	1.02 (0.818)
Lymphatic vessel invasion	Absent vs. present	1.18 (0.619)	-1.47 (0.119)	1.10 (0.878)	-1.03 (0.643)	-1.03 (0.914)
Perineural invasion	Absent vs. present	-4.11 (0.782)	1.00 (0.971)	1.36 (0.544)	-1.74 (0.686)	***1.37 (0.028)***
Clinical stage (TNM)	I vs. III	***2.40 (0.025)***	1.08 (0.642)	- 1.47 (0.869)	***1.30 (0.042)***	1.14 (0.348)

*FR*, Fold regulation; *Adeno.*, Adenocarcinoma. Significant values are bold.

### Analysis of genes of interest

After RNA extraction and cDNA synthesis, tumor samples from the 114 cases of CRC were analyzed by RT-PCR for the amplification of 84 ECM genes using the Extracellular Matrix and Adhesion Molecules PCR Array plate (PAHS-013; SABiosciences, Qiagen, Valencia, CA, USA). Of these 84 genes, *ITGAV*, *ITGA3*, *ITGA5*, *ITGB5*, *ITGA6* were selected. Each sample was analyzed using an ECM and adhesion molecule PCR array (PAHS-013; SABiosciences, Qiagen) plate. A mixture was prepared containing 1.275 ml of buffer with SYBR Green (2× Master Mix SABiosciences RT2 qPCR), 1.173 ml RNAse-free H_2_O and 102 μl of the cDNA sample. Next, 25-μl aliquots were added to each well of the 96-well plate. Reactions were performed in a thermal cycler (ABI 7500; Applied Biosystems, Foster City, CA, USA), according to the following protocol: 95°C for 10 min, and 40 cycles at 95°C for 15 s and 60°C for 1 min. Data analysis was performed using the ΔΔCt method from the website http://pcrdataanalysis.sabiosciences.com/pcr/arrayanalysis.php. Expression of each gene was classified as ‘high’ or ‘low’, based on the level of expression after grouping patients by the covariates of interest.

**Table 3:** Description of the tissue expression of integrins according to categorization of IHC expression by TMA technique (n = 114)

Integrin	Low expression		High expression	
	n	%	n	%
α3	55	48.2	59	51.8
α 6	61	53.5	53	46.5
α 5	53	46.5	61	53.5
α V	62	54.4	52	45.6
β 5	63	55.3	51	44,7

### Immunohistochemical assays

The immunohistochemical expression of proteins related to the selected genes was evaluated by TMA. The expression levels of the markers p53, Bcl-2, Ki67, epidermal growth factor receptor (EGFR) and vascular endothelial growth factor (VEGF) were also evaluated. Histological sections (4 μm thick) were stained with hematoxylin-eosin and reviewed, and the sites for TMA sampling were also selected. TMA blocks were prepared using Beecher apparatus (Beecher Instruments, Silver Spring, MD, USA), according to the manufacturer’s instructions. The TMA block sections were attached to the slides using an adhesive tape system (Instrumedics Inc., Hackensack, NJ, USA). The samples were cut to a thickness of 4 μm, and a small roller was used to press the section onto the tape. The tape with the attached histological section was then placed on a resin-coated slide (part of the adhesive system kit) and pressed with the same roller for better adherence. The slides were then placed under UV light for 20 min and were exposed to a solvent solution (TPC) for a further 20 min. The slides were dried, and the tape was removed. The sections were paraffin-embedded and stored in ideal cooling conditions.

Sections of TMA blocks were mounted onto glass slides coated with silane (3-aminopropyltriethoxysilane) and dried for 30 min at 37°C, deparaffinized with xylene and rehydrated through a series of graded alcohols. Endogenous peroxidase activity was blocked by incubating the sections in a bath of methanol containing 3% hydrogen peroxide for 20 min, followed by washing in distilled water. The sections were initially submitted to heat-induced epitope retrieval using citrate buffer (pH 9.0) in an uncovered pressure cooker (Eterna®, Nigro, Araraquara, Brazil). The slides were immersed in the buffer solution, and the pressure cooker was closed with the safety valve open; once the saturated steam was released, the safety valve was lowered until full pressurization was achieved. Endogenous peroxidase was blocked with 3% hydrogen peroxide (10 vol. hydrogen peroxide) for three washes of 10 min each. The slides were washed again in running distilled water, followed by 10 mM phosphate-buffered saline, pH 7.4, for 5 minutes. Primary antibodies were then applied, and the slides were incubated overnight at 8°C.

The following primary monoclonal antibodies were purchased from Abcam (Cambridge, MA, USA) and used at a 1:400 dilution: mouse anti-α6 integrin (100 μg), rabbit anti-β5 integrin (500 μl), mouse anti-α3 integrin (100 μg), mouse anti-αV integrin (100 μg), and mouse anti-α5 integrin (100 μl). The following non-ECM primary antibodies were also used: anti-p53 (1:300), anti-Bcl-2 (1:600), anti-VEGF (1:100), anti-Ki67 (1:500), and anti-EGFR (1:100).

### Specimen classification based on immunohistochemical results

Preliminary tests were performed to identify the optimal antibody concentrations and to select positive and negative controls using the dilution data supplied by the manufacturer.

After washing the primary antibody with phosphate-buffered saline, the slides were incubated with biotin-free polymer in the Advance ™ visualization system (DAKO) for 30 min. A freshly prepared solution containing 1 drop of DAB (3.3 - diaminobenzidine tetrahydrochloride; Sigma, St. Louis, Mo., USA) with 1 ml of substrate (DAKO) was applied for 5 min on each slide.

Tissue expression of markers was categorized dichotomously as either ‘over-expression’ or ‘under-expression’, according to the ‘quick score’ method [17, 18], which multiplies the percentage of stained cells (P) by the intensity of staining (I). The percentages of stained tumor cells were Scored as follows: 0 (absence of stained cells), 1 (<25% stained cells), 2 (26–50% stained cells) and 3 (>50% stained cells). Scores for the intensity of cell staining were as follows: 1 (mild intensity), 2 (moderate intensity) and 3 (intense staining). A gene product was thus considered to be over-expressed when the final score was >4 (P × I = >4), while markers with a final score ≤4 were considered to be under-expressed.

Stroma and tumor cells were not treated separately during immunohistochemical analysis, and only the expression levels of markers on tumor cells were considered for scoring (Figure 1).

### Statistical analysis

Data from real-time PCR were analyzed using the RT2 Profiler PCR Array Data Analysis program, version 3.4 (SABioscience, Qiagen) (http://pcrdataanalysis.sabiosciences.com/pcr/arrayanalysis.php). Statistical associations between integrin gene and protein expression levels and clinicopathological factors were determined using non-parametric Mann–Whitney U tests for quantitative variables and χ^2^ tests for qualitative variables. When the χ^2^ assumptions were not met, Fisher’s exact test was used.

**Table 4:** Analysis of IHC expression of Integrins expression according to covariates in patients with CRC (n = 114)

Covariates	Categorization	Integrins									
		αV		α3		α5		α6		β5	
		- / +	p	- / +	p	- / +	p	- / +	p	- / +	p
Age	< 60 years	27/29	0.259	27/29	1.00	**49/7**	***<0.001***	30/26	1.000	30/26	0.851
	≥ 60 years	35/23		28/30		**4/54**		31/27		33/25	
Gender	Male	34/29	1.000	28/35	0.451	29/34	1.000	36/27	0.452	35/28	1.00
	Female	28/23		27/24		24/27		25/26		28/23	
Histological type	Adeno	54/43	0.601	48/49	0.502	**51/ 46**	***0.003***	**61/36**	***0.002***	53/44	0.684
	Mucinous	8/9		7/10		**2/15**		**0/17**		10/7	
Tumor grading	Low grade	56/44	0.401	50/50	0.397	48/52	0.569	57/43	0.083	57/43	0.394
	High grade	6/8		5/9		5/9		4/10		6/8	
Venous invasion	Absent	**58/35**	***<0.001***	**52/41**	***0.001***	42/51	0.631	**61/32**	***<0.001***	54/39	0.239
	Present	**4/17**		**3/18**		11/10		0**/21**		09/12	
Perineural invasion	Absent	**62/44**	***0.001***	52/54	0.781	49/57	1.000	59/47	0.142	60/46	0.464
	Present	**0/8**		3/5		4/4		2/6		3/5	
Clinical stage	I-II	**59/5**	***<0.001***	**53/11**	***<0.001***	28/36	0.572	**40/24**	***0.038***	**49/15**	***<0.001***
	III- IV	**3/47**		**2/48**		25/25		**21/29**		**14/36**	

-/+ = Low/high expression; *Adeno.*,Adenocarcinoma. Significant values are bold.

The associations between integrin genes and the non-ECM markers EGFR, VEGF, p53, Bcl-2 and Ki67 (ordinal variables) were measured using the Spearman correlation coefficient. The Spearman coefficient may range from -1 to +1, and the closer the calculated value is to these extremes (-1 or +1), the greater the association between the variables [19].

The level of significance was set at 5% (*P* < 0.05), and the data were analyzed using SPSS software (Statistical Package for Social Sciences; SPSS, Chicago, IL, USA), version 15.0. The Shapiro-Wilk test was used to verify that the data were normally distributed.

## Results

### Integrin gene expression in colon cancer tissues analyzed by RT-PCR

The *ITGA3* gene was significantly over-expressed in TNM III tumors compared with TNM I tumors (2.40 -fold regulation; *P* = 0.025). *ITGA5* was over-expressed in histological mucinous type compared with adenocarcinomas (1.25-fold regulation; *P* = 0.029), and under-expressed in patients aged over 60 years, compared with those under 60 (1.54-fold regulation; *P* = 0.016). The *ITGB5* gene was over-expressed in TNM III compared with TNM I stages (1.30-fold regulation; *P* = 0.042). The *ITGA6* gene was over-expressed in tumors with venous invasion compared with those without (1.42-fold regulation; *P* = 0.047), while the *ITGAV* gene was over-expressed in tumors with perineural invasion, compared with those without (1.37-fold regulation; *P* = 0.02). Regarding the degree of cell differentiation, there were no significant differences in expression levels of gene (grades I-II compared with grade III) . A summary of these results is shown in Table 2.

**Table 5:** Expression levels of integrins genes in relation to clinicopathological variables and immunohistochemical scores (p < 0.05)

Integrin	Gene expression - group	Protein expression - group	Validation
α6	Over - vascular invasion +	Over - vascular invasion +	Yes
		Over - mucinous	No
		Over - TNM III,IV	No
αV	Over - neural invasion +	Over - neural invasion +	Yes
		Over - TNM III,IV	No
		Over - vascular invasion +	No
α5	Over – mucinous	Over - mucinous	Yes
	Under - age >60	Over – age >60	No
β5	Over - TNM III	Over - TNM III,IV	Yes
α3	Over - TNM III	Over - TNM III,IV	Yes
		Over- vascular invasion +	No

### Immunohistochemical study of integrins in colon cancer tissues

Table 3 shows the frequencies of high and low expression of the gene products of interest for the 114 patients included in this study.

Regarding the degree of cell differentiation, there were no significant differences in expression levels of any proteins between tumors scored as immunohistochemical grades I-II compared with grade III (*P* > 0.05). In terms of TNM staging, however, ITGB5, ITGAV, ITGA3 and ITGA6 proteins were significantly over-expressed in stages III and IV compared with stages I and II (*P* <0.05).

In relation to the presence of peritumoral inflammatory infiltrate, there were no significant differences in expression levels of any of the evaluated proteins (*P* > 0.05) in relation to the presence or absence of inflammatory infiltrate. Regarding the presence vs. the absence of venous invasion, however, ITGAV, ITGA3 and ITGA6 were significantly over-expressed in the presence of venous invasion (*P* < 0.05). In addition, ITGAV was significantly over-expressed in tumors showing perineural invasion (*P* < 0.05), and ITGA5 and ITGA6 were significantly over-expressed in mucinous-type tumors compared with adenocarcinoma (*P* < 0.05).

There were no significant differences in expression levels between genders for any of the analyzed proteins (*P* > 0.05). However, ITGA5 protein was over-expressed in patients under 60 years old compared with those over 60 years (*P* < 0.05). Table 4 shows the results of immunoexpression of these markers according to the clinicopathological covariates studied.

For each integrin gene that was under- or over-expressed according to array tracking, the corresponding protein was analyzed by antigen-antibody reaction on TMA slides. Protein expression levels validated the RT-PCR results, with the exception of *ITGA5* expression in relation to age. A summary of these results is presented in Table 5.

### Relationship between integrin expression and epithelial markers

The associations between integrin genes and the epithelial markers EGFR, VEGF, p53, Bcl-2 and Ki67 were analyzed using the Spearman correlation coefficient. Significant associations were found between *ITGAV*/EGFR (r = 0.774; *P* < 0.001), *ITGA3*/EGFR (r = 0.744; *P* < 0.001) and p53/Ki67 (r = 0.875; *P* < 0.001).

The Spearman correlation is presented in Table 6.

**Table 6:** Spearman coefficient correlation (r, two-tailed model) for associations between IHC expression of integrins proteins and other markers (n = 114)

		ITGB5	ITGA5	ITGAV	ITGA6	ITGA3	EGFR	VEGF	KI67	P53	BCL2
**ITGB5**	ρ	1,000	0,025	0,380	0,116	0,480	0,428	-0,049	0,104	0,057	-0,018
	Valor de p	.	0,791	0,000*	0,218	0,000*	0,000*	0,603	0,270	0,548	0,852
**ITGA5**	ρ	0,025	1,000	-0,100	0,199	-0,020	-0,003	0,086	-0,205	-0,193	0,018
	Valor de p	0,791	.	0,291	0,034*	0,832	0,976	0,366	0,028*	0,040*	0,853
**ITGAV**	ρ	0,380	-0,100	1,000	0,135	0,708	0,774	-0,103	0,262	0,215	-0,106
	Valor de p	0,000*	0,291	.	0,152	0,000*	0,000*	0,277	0,005*	0,022*	0,263
**ITGA6**	ρ	0,116	0,199	0,135	1,000	0,231	0,219	0,055	0,134	0,086	-0,088
	Valor de p	0,218	0,034*	0,152	.	0,013*	0,019*	0,559	0,154	0,366	0,352
**ITGA3**	ρ	0,480	-0,020	0,708	0,231	1,000	0,744	0,051	0,155	0,100	0,018
	Valor de p	0,000*	0,832	0,000*	0,013*	.	0,000*	0,587	0,101	0,288	0,853
**EGFR**	ρ	0,428	-0,003	0,774	0,219	0,744	1,000	-0,082	0,375	0,335	-0,054
	Valor de p	0,000*	0,976	0,000*	0,019*	0,000*	.	0,384	0,000*	0,000*	0,569
**VEGF**	ρ	-0,049	0,086	-0,103	0,055	0,051	-0,082	1,000	-0,165	-0,150	0,334
	Valor de p	0,603	0,366	0,277	0,559	0,587	0,384	.	0,080	0,111	0,000*
**KI67**	ρ	0,104	-0,205	0,262	0,134	0,155	0,375	-0,165	1,000	0,875	-0,124
	Valor de p	0,270	0,028	0,005*	0,154	0,101	0,000*	0,080	.	0,000*	0,189
**P53**	ρ	0,057	-0,193	0,215	0,086	0,100	0,335	-0,150	0,875	1,000	-0,107
	Valor de p	0,548	0,040*	0,022*	0,366	0,288	0,000*	0,111	0,000*	.	0,256
**BCL2**	ρ	-0,018	0,018	-0,106	-0,088	0,018	-0,054	0,334	-0,124	-0,107	1,000
	Valor de p	0,852	0,853	0,263	0,352	0,853	0,569	0,000*	0,189	0,256	.

## Discussion

There is considerable evidence to implicate genetic alterations in the rapid progression of several types of malignant tumors from the early to more advanced stages. Abnormal signaling of molecules may activate genes and thus trigger dissemination and metastasis. The identification of these altered molecules and their correlations with clinical and pathological stages may help to elucidate the mechanisms involved in this processes.

Koivisto et al. [20] suggested that the ECM has a decisive influence on tumor behavior, especially in processes of proliferation, progression and tumor cell invasion. These interactions are mediated by integrins, which play an important role in the development of tumor invasion and metastasis. This study highlighted the roles of the integrin membrane receptors, which are the most-studied and well-understood cell adhesion molecules [4, 8, 21]. The extracellular portion of the integrin is known to bind to ECM proteins, while the intracellular portion connects to cytoskeletal elements such as actin filaments. This connection reinforces the integrity of tissues and cell adhesion, and stabilizes cellular protrusions during migration. This connection also represents a signaling pathway that can transmit information to key processes such as transcriptional control, cell death, proliferation and migration [22]. Furthermore, integrins have been shown to be differentially expressed during tumor growth and progression, making them potential targets for the diagnosis and therapy of cancer [14–16, 23].

In this study, we detected over-expression of the genes for α3 and β5 integrins in more advanced tumors, in stages III compared with stage I, which represent non-metastatic tumors. This observation was confirmed by TMA protein analysis, suggesting a relationship between these integrins and tumor progression and dissemination. According to Jinka et al. [24], over-expression of integrins α3, α5 and α6 was directly related to the progression of various types of malignant tumors. Haier et al. [25] studied the expression of α2, α3, α5 and α6 integrins by immunohistochemistry in cell lineages from metastatic colorectal liver carcinoma, and showed over-expression of α2 and α3 integrin in relation to dissemination potential. Another immunohistochemical study by Toquet et al. showed higher expression of α5 integrin in poorly differentiated cells in grade-III tumors, compared with grades I and II [26]. This study demonstrated a significant relationship between α5 integrin expression and mucinous histological type vs. adenocarcinoma, the latter of which has a better prognosis.

A recent cell-culture study of human breast cancer and normal epithelial tissue showed an involvement of β5 integrin in tumor progression and invasion in terms of altered adhesion, cell architecture, and differentiation, and noted that inhibition of this integrin significantly reduced breast carcinoma cell invasion [27]. α6 Integrin regulates multiple cellular functions, including the development of cell invasion, migration and tumor progression [28]. However, to the best of our knowledge, the current study is the first to demonstrate a correlation between α6 integrin gene over-expression and venous invasion, thus connecting tumor spread with hyper-expression of this integrin. Further studies are needed to confirm these findings. A recent study [29] examined breast cancer cell lineages in rats by RT-PCR and flow cytometry, and concluded that α6 integrin worked as a promoter for cell metastasis and accelerated cell proliferation, indicating its involvement in tumor progression.

Neural invasion was associated with a significantly lower survival rate and an increased recurrence rate in patients with rectal cancer stage III and IV [30]. In the present study, expression of the *ITGAV* gene was significantly related to the presence of perineural invasion (*P* = 0.02), as confirmed by TMA analysis. Although some integrin subtypes have been shown to be associated with perineural invasion in prostate cancer [31] and carcinomas of the head and neck [32], no previous study has demonstrated a relationship between over-expression of *ITGAV* and the presence of perineural invasion in CRC.

We also examined the associations between integrin protein expression and expression of selected epithelial markers. EGFR showed a strong correlation with αV integrin and a moderate correlation with α3 integrin (both *P* < 0.05). Other studies have suggested that integrins may also modulate the intracellular recycling of growth factor receptors such as EGFR [33] and VEGFR [34]. Other authors reported that the EGFR-integrin interaction seen in pancreatic cancer also increased the migration of colon cancer cells through the integrins α3β1 and α6β4, and acted in hepatocellular carcinoma through integrins α1β1 and α2β1 [35, 36].

## Conclusions

Increased expression levels of *ITGA6* and *ITGAV* are related to venous invasion and neural infiltration, respectively, while over-expression of *ITGB5* and *ITGA3* are associated with stage III (TNM), and over-expression of *ITGA5* correlates with the presence of mucinous-type malignant neoplasias.

Further follow-up studies, preferably with a controlled prospective design, are necessary to establish the roles of integrins as potential biomarkers that could predict disease extent or outcome, and possibly contribute to the management of patients with CRC.

## Authors’ original submitted files for images

Below are the links to the authors’ original submitted files for images. Authors’ original file for figure 1

## References

[1] Guest I, Uetrecht J: Drugs that induce neutropenia/agranulocytosis may target specific components of the stromal cell extracellular matrix. Med Hypotheses. 1999, 53 (2): 145-151. 10.1054/mehy.1998.0734. 10532710 10.1054/mehy.1998.0734

[2] Kram V, Zcharia E, Yacoby-Zeevi O, Metzger S, Chajek-Shaul T, Gabet Y: Heparanase is expressed in osteoblastic cells and stimulates bone formation and bone mass. J Cell Physiol. 2006, 207 (3): 784-792. 10.1002/jcp.20625. 16514606 10.1002/jcp.20625

[3] Milner R, Campbell IL: The integrin family of cell adhesion molecules has multiple functions within the CNS. J Neurosci Res. 2002, 69 (3): 286-291. 10.1002/jnr.10321. 12125070 10.1002/jnr.10321

[4] Hynes RO: Integrins: bidirectional, allosteric signaling machines. Cell. 2002, 110 (6): 673-687. 10.1016/S0092-8674(02)00971-6. 12297042 10.1016/s0092-8674(02)00971-6

[5] Humphries JD, Byron A, Humphries MJ: Integrin ligands at a glance. J Cell Sci. 2006, 119 (Pt 19): 3901-3903. 16988024 10.1242/jcs.03098 PMC3380273

[6] Labat-Robert J: Fibronectin in malignancy. Semin Cancer Biol. 2002, 12 (3): 187-195. 10.1016/S1044-579X(02)00022-6. 12083849 10.1016/S1044-579X(02)00022-6

[7] Thomas GJ, Jones J, Speight PM: Integrins and oral cancer. Oral Oncol. 1997, 33 (6): 381-388. 10.1016/S0964-1955(97)00021-3. 9509120 10.1016/s0964-1955(97)00021-3

[8] Hynes RO: Integrins: a family of cell surface receptors. Cell. 1987, 48 (4): 549-554. 10.1016/0092-8674(87)90233-9. 3028640 10.1016/0092-8674(87)90233-9

[9] Park CC, Bissell MJ, Barcellos-Hoff MH: The influence of the microenvironment on the malignant phenotype. Mol Med Today. 2000, 6 (8): 324-329. 10.1016/S1357-4310(00)01756-1. 10904250 10.1016/s1357-4310(00)01756-1

[10] Brown EJ: Integrin-associated proteins. Curr Opin Cell Biol. 2002, 14 (5): 603-607. 10.1016/S0955-0674(02)00360-5. 12231356 10.1016/s0955-0674(02)00360-5

[11] Zhang Y, Lu H, Dazin P, Kapila Y: Squamous cell carcinoma cell aggregates escape suspension-induced, p53-mediated anoikis: fibronectin and integrin alphav mediate survival signals through focal adhesion kinase. J Biol Chem. 2004, 279 (46): 48342-48349. 10.1074/jbc.M407953200. 15331608 10.1074/jbc.M407953200

[12] von Lampe B, Stallmach A, Riecken EO: Altered glycosylation of integrin adhesion molecules in colorectal cancer cells and decreased adhesion to the extracellular matrix. Gut. 1993, 34 (6): 829-836. 10.1136/gut.34.6.829. 8314518 10.1136/gut.34.6.829 PMC1374271

[13] Ahmed N, Niu J, Dorahy DJ, Gu X, Andrews S, Meldrum CJ: Direct integrin alphavbeta6-ERK binding: implications for tumour growth. Oncogene. 2002, 21 (9): 1370-1380. 10.1038/sj.onc.1205286. 11857080 10.1038/sj.onc.1205286

[14] Avraamides CJ, Garmy-Susini B, Varner JA: Integrins in angiogenesis and lymphangiogenesis. Nat Rev Cancer. 2008, 8 (8): 604-617. 10.1038/nrc2353. 18497750 10.1038/nrc2353 PMC2577722

[15] Giancotti FG, Tarone G: Positional control of cell fate through joint integrin/receptor protein kinase signaling. Annu Rev Cell Dev Biol. 2003, 19: 173-206. 10.1146/annurev.cellbio.19.031103.133334. 14570568 10.1146/annurev.cellbio.19.031103.133334

[16] Hwang R, Varner J: The role of integrins in tumor angiogenesis. Hematol Oncol Clin North Am. 2004, 18 (5): 991-1006. 10.1016/j.hoc.2004.09.010. vii 15474331 10.1016/j.hoc.2004.09.010

[17] Hoos A, Cordon-Cardo C: Tissue microarray profiling of cancer specimens and cell lines: opportunities and limitations. Lab Invest. 2001, 81 (10): 1331-1338. 10.1038/labinvest.3780347. 11598146 10.1038/labinvest.3780347

[18] Bertucci F, Salas S, Eysteries S, Nasser V, Finetti P, Ginestier C: Gene expression profiling of colon cancer by DNA microarrays and correlation with histoclinical parameters. Oncogene. 2004, 23 (7): 1377-1391. 10.1038/sj.onc.1207262. 14973550 10.1038/sj.onc.1207262

[19] Spearman C: The proof and measurement of association between two things. Int J Epidemiol. 2010, 39 (5): 1137-1150. 10.1093/ije/dyq191. 21051364 10.1093/ije/dyq191

[20] Koivisto L, Grenman R, Heino J, Larjava H: Integrins alpha5beta1, alphavbeta1, and alphavbeta6 collaborate in squamous carcinoma cell spreading and migration on fibronectin. Exp Cell Res. 2000, 255 (1): 10-17. 10.1006/excr.1999.4769. 10666329 10.1006/excr.1999.4769

[21] Miranti CK, Brugge JS: Sensing the environment: a historical perspective on integrin signal transduction. Nat Cell Biol. 2002, 4 (4): E83-E90. 10.1038/ncb0402-e83. 11944041 10.1038/ncb0402-e83

[22] Ulrich F, Heisenberg CP: Trafficking and cell migration. Traffic. 2009, 10 (7): 811-818. 10.1111/j.1600-0854.2009.00929.x. 19490534 10.1111/j.1600-0854.2009.00929.x

[23] Mizejewski GJ: Role of integrins in cancer: survey of expression patterns. Proc Soc Exp Biol Med. 1999, 222 (2): 124-138. 10.1046/j.1525-1373.1999.d01-122.x. 10564536 10.1177/153537029922200203

[24] Jinka R, Kapoor R, Sistla PG, Raj TA, Pande G: Alterations in cell-extracellular matrix interactions during progression of cancers. Int J Cell Biol. 2012, 2012: 8-10.1155/2012/219196. Article ID 219196, http://www.hindawi.com/journals/ijcb/2012/219196/10.1155/2012/219196 PMC3259478 22262973

[25] Haier J, Nasralla M, Nicolson GL: Different adhesion properties of highly and poorly metastatic HT-29 colon carcinoma cells with extracellular matrix components: role of integrin expression and cytoskeletal components. Br J Cancer. 1999, 80 (12): 1867-1874. 10.1038/sj.bjc.6690614. 10471033 10.1038/sj.bjc.6690614 PMC2374274

[26] Toquet C, Colson A, Jarry A, Bezieau S, Volteau C, Boisseau P: ADAM15 to alpha5beta1 integrin switch in colon carcinoma cells: a late event in cancer progression associated with tumor dedifferentiation and poor prognosis. Int J Cancer. 2012, 130 (2): 278-287. 10.1002/ijc.25891. Epub 2011 Nov 9 21190186 10.1002/ijc.25891

[27] Bianchi A, Gervasi ME, Bakin A: Role of beta5-integrin in epithelial-mesenchymal transition in response to TGF-beta. Cell Cycle. 2010, 9 (8): 1647-1659. 10.4161/cc.9.8.11517. 20404485 10.4161/cc.9.8.11517

[28] Guo W, Giancotti FG: Integrin signalling during tumour progression. Nat Rev Mol Cell Biol. 2004, 5 (10): 816-826. 10.1038/nrm1490. 15459662 10.1038/nrm1490

[29] Wang Y, Shenouda S, Baranwal S, Rathinam R, Jain P, Bao L: Integrin subunits alpha5 and alpha6 regulate cell cycle by modulating the chk1 and Rb/E2F pathways to affect breast cancer metastasis. Mol Cancer. 2011, 10: 84-10.1186/1476-4598-10-84. PMID: 21752283 [PubMed - indexed for MEDLINE] 21752283 10.1186/1476-4598-10-84 PMC3163626

[30] Ceyhan GO, Liebl F, Maak M, Schuster T, Becker K, Langer R: The severity of neural invasion is a crucial prognostic factor in rectal cancer independent of neoadjuvant radiochemotherapy. Ann Surg. 2010, 252 (5): 797-804. 10.1097/SLA.0b013e3181fcab8d. 21037435 10.1097/SLA.0b013e3181fcab8d

[31] Sroka IC, Anderson TA, McDaniel KM, Nagle RB, Gretzer MB, Cress AE: The laminin binding integrin alpha6beta1 in prostate cancer perineural invasion. J Cell Physiol. 2010, 224 (2): 283-288. 10.1002/jcp.22149. 20432448 10.1002/jcp.22149 PMC4816210

[32] Dyce OH, Ziober AF, Weber RS, Miyazaki K, Khariwala SS, Feldman M: Integrins in head and neck squamous cell carcinoma invasion. Laryngoscope. 2002, 112 (11): 2025-2032. 10.1097/00005537-200211000-00021. 12439174 10.1097/00005537-200211000-00021

[33] Caswell PT, Chan M, Lindsay AJ, McCaffrey MW, Boettiger D, Norman JC: Rab-coupling protein coordinates recycling of alpha5beta1 integrin and EGFR1 to promote cell migration in 3D microenvironments. J Cell Biol. 2008, 183 (1): 143-155. 10.1083/jcb.200804140. 18838556 10.1083/jcb.200804140 PMC2557049

[34] Reynolds AR, Hart IR, Watson AR, Welti JC, Silva RG, Robinson SD: Stimulation of tumor growth and angiogenesis by low concentrations of RGD-mimetic integrin inhibitors. Nat Med. 2009, 15 (4): 392-400. 10.1038/nm.1941. 19305413 10.1038/nm.1941

[35] Pouliot N, Nice EC, Burgess AW: Laminin-10 mediates basal and EGF-stimulated motility of human colon carcinoma cells via alpha[3]beta[1] and alpha[6]beta[4] integrins. Exp Cell Res. 2001, 266 (1): 1-10. 10.1006/excr.2001.5197. 11339819 10.1006/excr.2001.5197

[36] Yang C, Zeisberg M, Lively JC, Nyberg P, Afdhal N, Kalluri R: Integrin alpha1beta1 and alpha2beta1 are the key regulators of hepatocarcinoma cell invasion across the fibrotic matrix microenvironment. Cancer Res. 2003, 63 (23): 8312-8317. 14678990

[CR37] The pre-publication history for this paper can be accessed here:http://www.biomedcentral.com/1472-6890/13/16/prepub

