# Supplementary figures and images for: RETRACTED ARTICLE: Expression of integrin genes and proteins in progression and dissemination of colorectal adenocarcinoma
Source: BMC Clin Pathol. 2013 May 24;13:16. doi: 10.1186/1472-6890-13-16 (PMC3671190; doi:10.1186/1472-6890-13-16)

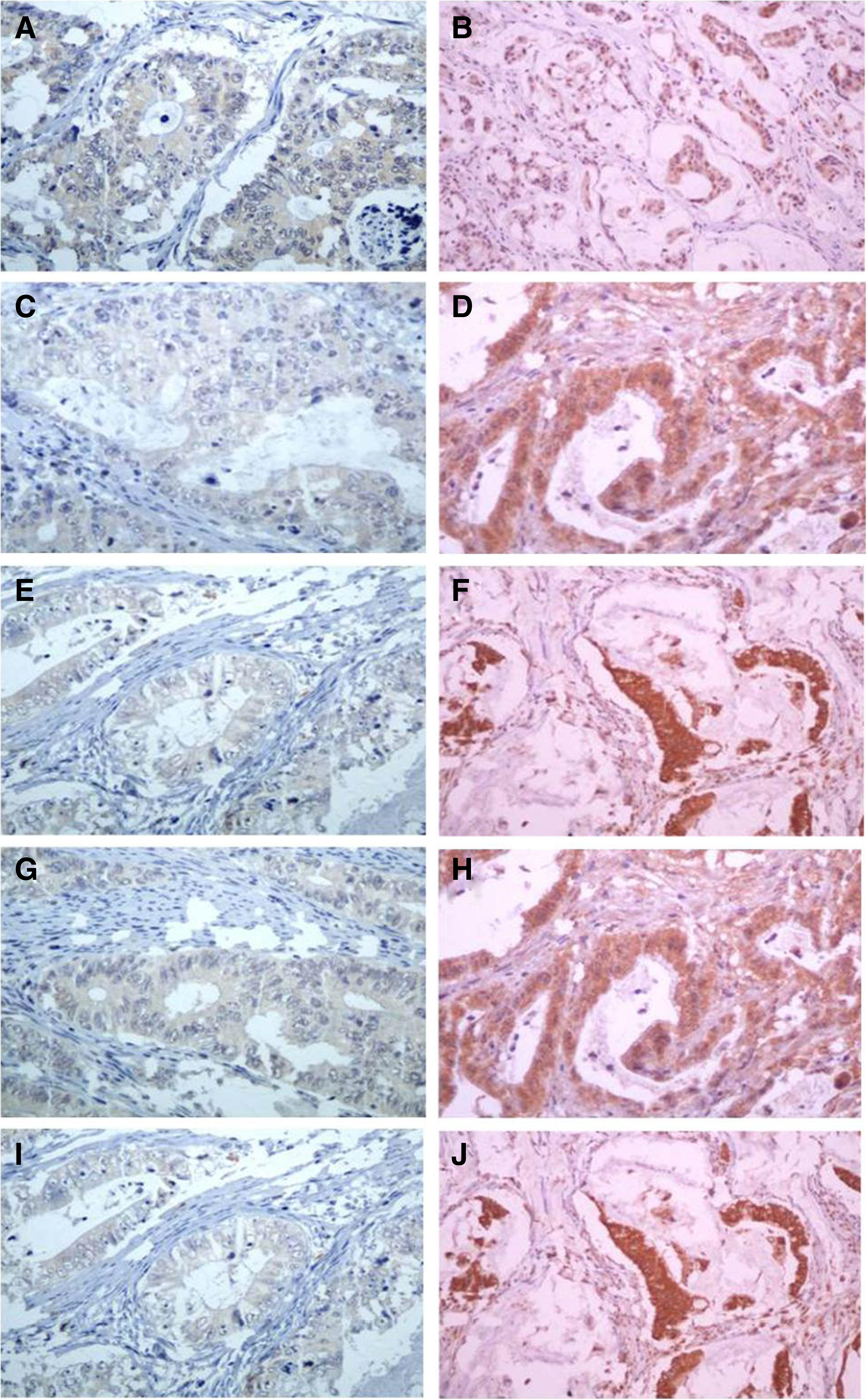

Supplement: Supplementary file 1 — Authors’ original file for figure 1 [file 12907_2012_134_MOESM1_ESM.tiff]
